# Evaluation of State-Mandated Reporting of Neonatal Abstinence Syndrome — Six States, 2013–2017

**DOI:** 10.15585/mmwr.mm6801a2

**Published:** 2019-01-11

**Authors:** Shahla M. Jilani, Meghan T. Frey, Dawn Pepin, Tracey Jewell, Melissa Jordan,, Angela M. Miller, Meagan Robinson, Tomi St. Mars, Michael Bryan, Jean Y. Ko, Elizabeth C. Ailes, Russell F. McCord, Julie Gilchrist, Sarah Foster, Jennifer N. Lind, Lindsay Culp, Matthew S. Penn, Jennita Reefhuis

**Affiliations:** ^1^Office of the Secretary, U.S. Department of Health and Human Services; ^2^National Center on Birth Defects and Developmental Disabilities, CDC; ^3^Cherokee Nation Assurance, Arlington, Virginia; ^4^Center for State, Tribal, Local, and Territorial Support, CDC; ^5^Kentucky Department for Public Health; ^6^Florida Department of Health; ^7^Tennessee State Department of Health; ^8^Virginia Department of Health; ^9^Arizona State Department of Health; ^10^Georgia Department of Public Health; ^11^National Center for Chronic Disease Prevention and Health Promotion, CDC; ^12^Oak Ridge Institute for Science and Education, Oak Ridge, Tennessee; ^13^Carter Consulting, Inc., Atlanta, Georgia; ^14^National Center for Injury Prevention and Control, CDC

From 2004 to 2014, the incidence of neonatal abstinence syndrome (NAS) in the United States increased 433%, from 1.5 to 8.0 per 1,000 hospital births. The latest national data from 2014 indicate that one baby was born with signs of NAS every 15 minutes in the United States (*1*). NAS is a drug withdrawal syndrome that most commonly occurs among infants after in utero exposure to opioids, although other substances have also been associated with NAS. Prenatal opioid exposure has also been associated with poor fetal growth, preterm birth, stillbirth, and possible specific birth defects (*2*–*5*). NAS surveillance has often depended on hospital discharge data, which historically underestimate the incidence of NAS and are not available in real time, thus limiting states’ ability to quickly direct public health resources (*6*,*7*). This evaluation focused on six states with state laws implementing required NAS case reporting for public health surveillance during 2013–2017 and reviews implementation of the laws, state officials’ reports of data quality before and after laws were passed, and advantages and challenges of legally mandating NAS reporting for public health surveillance in the absence of a national case definition. Using standardized search terms in an online legal research database, laws in six states mandating reporting of NAS from medical facilities to state health departments (SHDs) or from SHDs to a state legislative body were identified. SHD officials in these six states completed a questionnaire followed by a semistructured telephone interview to clarify open-text responses from the questionnaire. Variability was found in the type and number of surveillance data elements reported and in how states used NAS surveillance data. Following implementation, five states with identified laws reported receiving NAS case reports within 30 days of diagnosis. Mandated NAS case reporting allowed SHDs to quantify the incidence of NAS in their states and to inform programs and services. This information might be useful to states considering implementing mandatory NAS surveillance.

To identify states with laws mandating reporting of NAS for public health surveillance, relevant laws (statutes and regulations) were identified using Westlaw,* an online legal research database, on January 3, 2018. Search terms were limited to identify statutes and regulations that explicitly named “neonatal abstinence syndrome” in states’ disease and conditions reporting laws. The search string was applied to all 50 states and the District of Columbia. Laws were cross-referenced with states’ disease reporting lists on SHD websites. Six states (Arizona, Florida, Georgia, Kentucky, Tennessee, and Virginia) were identified as having laws requiring reporting of NAS from medical facilities to the SHD, from the SHD to a state legislative body, or both. SHD officials in these six states completed a 28-item questionnaire, and a semistructured telephone interview (focusing on development of statute, implementation, data collection and quality) was conducted with one interviewee per state. Interviewees were identified via outreach to SHD officials requesting SHD points of contact for, or designated experts on, NAS surveillance. Questionnaire and interview data were analyzed for similarities and differences in NAS reporting criteria, data elements and utilization, reporting system, required resources, and barriers to case reporting.

A review of the six states’ laws indicated variation in states’ reporting frameworks (Table 1). Laws in Arizona, Florida, Georgia, Kentucky, and Virginia require medical providers and medical facilities to report cases of NAS to their respective SHDs. In Tennessee, the health commissioner has the authority to add new diseases to the reportable disease list without a new statute or regulation. Using this authority, NAS was made reportable from medical facilities to the Tennessee SHD without a new law in 2013; therefore, the 2013 implementation is not included in this review of NAS laws. However, Tennessee’s 2017 law, which explicitly names “neonatal abstinence syndrome,” was captured in the Westlaw search; therefore, the 2017 law requiring the SHD to report NAS cases to the Tennessee state legislature was included in this analysis. Georgia’s 2017 law also requires any medical provider who has diagnosed an infant with NAS to report the case to the SHD and the SHD to report cases to the state legislature. Georgia’s and Virginia’s laws define NAS, whereas the other four states’ laws do not. Arizona’s law specifies data elements to be collected. State laws vary in the required time frame for case reporting from “at the time of diagnosis” to within 6 months after diagnosis.

**TABLE 1:** Legislation mandating neonatal abstinence syndrome (NAS) case reporting — six states, 2013–2017

State	Citation	Effective year	Is there a definition of NAS used in the law?	Who must report NAS?		To whom must NAS be reported?		Time frame for reporting to	
				Provider/Facility*	Dept. of Health	Dept. of Health	Legislative body	Dept. of Health	Legislative body
Arizona	AZ. Admin. code ^§^ R9–4-602	2017	No	Yes	—	Yes	—	5 business days	N/A
Florida	FL. Admin. Code Ann. r. 64D-3.029	2014	No	Yes	—	Yes	—	6 months^†^	N/A
Georgia	GA. Code Ann. ^§^ 31–12–2	2017	Yes^§^	Yes	Yes^¶^	Yes	Yes	N/A**	annually
Kentucky	KY. Rev. Stat. Ann. ^§^ 211.676	2013	No	Yes	—	Yes	—	at time of diagnosis	N/A
	KY. Rev. Stat. Ann. ^§^ 211.678	2014							
Tennessee^††^	TN. Code Ann. ^§^ 68–1-805	2017	No	—	Yes	—	Yes	N/A	annually
Virginia^§§^	12 VA. Admin. Code ^§^ 5–90–80^¶¶^	2017	Yes***	Yes	—	Yes	—	1 month	N/A

**Abbreviations:** AZ = Arizona; FL = Florida; GA = Georgia; KY = Kentucky; N/A = not applicable; TN = Tennessee; VA = Virginia.

* Defines providers broadly to include coroners and medical examiners. Facilities are also defined broadly to include hospitals, birthing centers, and various healthcare facilities. Individual states might have laws with additional mandatory reporters. For example, see GA. Code Ann. ^§^ 31–12–2, in which “any other person or entity the department determines has knowledge of diagnosis or health outcomes related, directly or indirectly” must also report NAS.

^†^ FL. Admin. Code Ann. r. 64D-3.029(3), FN 18. Within 6 months, hospitals must “report each case of neonatal abstinence syndrome occurring in an infant admitted to the hospital.” However, “[i]f a hospital reports a case of neonatal abstinence syndrome to the Agency for Health Care Administration in its inpatient discharge data report, pursuant to Chapter 59E-7, F.A.C., then it need not comply with the reporting requirements of subsection 64D-3.029(1), F.A.C.”

^§^ GA. Code Ann. ^§^ 31–12–2. “’[N]eonatal abstinence syndrome’ means a group of physical problems that occur in a newborn infant who was exposed to addictive illegal or prescription drugs while in the mother’s womb.”

^¶^ The Georgia Department of Health must report NAS case load and NAS incidence to the state legislature on a yearly basis.

** GA. Code Ann. ^§^ 31–12–2 indicates that reporting shall take place “in a manner and at such times as may be prescribed.” The health department has used this authority to require a 30-day time frame for reporting.

^††^ See also Tenn. Comp. R. and Regs. 1200–14–01-.02 (2010). This law does not use the terminology “neonatal abstinence syndrome” but does authorize the health commissioner to add diseases to the reportable disease list, which requires providers to report to the state health department. Tennessee added NAS to its reportable disease list in 2013.

^§§^ See also VA Code Ann. ^§^ 32.1–35 (West 2018). This law does not use the terminology “neonatal abstinence syndrome” but does authorize the board to add diseases to the reportable disease list. NAS is on the reportable disease list in Virginia.

^¶¶^ Virginia’s legislature enacted an uncodified act (SB1323/HB1467) Acts 2017, mL. 185 and 280, requiring the Board of Health to adopt regulation to include NAS as a reportable disease.

*** 12 VA Admin Code ^§^ 5–90–80. “[A] condition characterized by clinical signs of withdrawal from exposure to prescribed or illicit drugs.”

The questionnaire and telephone interviews were completed during March–May 2018. All six states identify reportable NAS cases based on a clinical diagnosis of NAS by a medical provider (Table 2). Georgia’s SHD also requires that infants with positive toxicology results be reported to the SHD as a NAS case even in the absence of a clinical diagnosis of NAS by a medical provider. Including positive infant toxicology results in Georgia’s NAS case definition allows the state to determine the types of substances infants are exposed to prenatally that might cause signs of withdrawal postnatally. Documented maternal opioid use is not a criterion for case reporting in any of the six states. None of these states reported administration of specific care or pharmacologic treatment to an infant as a criterion for case reporting. Health officials in Kentucky commented that they do not define cases based on an abstinence scoring tool (*8*,*9*) because of potential subjective differences in how providers quantify symptoms as part of the scoring method. During interviews, state officials consistently noted that mandated reporting of NAS was enacted to 1) gain a more precise understanding of the incidence of NAS in their state, 2) better characterize the impact of the opioid crisis in their state, 3) identify specific communities or geographic areas more severely affected by opioids and NAS, and 4) inform programs and services.

**TABLE 2:** Advantages and challenges of surveillance features reported by health officials among states with mandated reporting of neonatal abstinence syndrome (NAS) — six states, 2013–2017

Surveillance feature reported in 28-item questionnaire	States endorsing surveillance feature in questionnaire	Advantages (+) and challenges (-) reported by health officials in open-text fields in questionnaire and during semistructured interviews
**Criteria for reporting NAS**		
Clinical diagnoses by medical provider*	AZ, FL, GA, KY, TN, VA	– Requires additional review to identify duplicate NAS cases (i.e., if infant is treated at multiple facilities or at delivery and at another encounter postdischarge)
		– Providers might look to state health departments for a case definition
		– Will not identify asymptomatic infants with prenatal substance exposure
		– Transition from *International Classification of Diseases Clinical Modification* (ICD)-9 to ICD-10 codes might affect the number and trends of cases identified in administrative data sets and require additional educational resources
Positive toxicology result for infant	GA^†^	+ Toxicology results allow state to determine whether substance exposure was from a prescribed medication or an illicit substance^§^
**Data elements collected in case reports**		
Maternal demographics	FL, GA, KY, TN	+ Allows for characterizations of populations at higher risk and areas of higher risk
Infant demographics	AZ, FL, GA, KY, TN, VA	+ Opportunity to identify patterns in specific geographic areas
Maternal source of exposure(s)	AZ, GA, KY, TN, VA	+ Can identify prenatal exposures
		+ Allows for comparison between clinical symptoms of withdrawal and substance exposure in the absence of clinical symptoms of withdrawal
		+ Provides information on polysubstance exposures
Heath care service utilization by infant	GA	+ Ability to estimate costs associated with treatment
		+ Can capture characteristics of treatment (e.g., length of stay)
Other	AZ, GA, KY, TN	+ Some variables (e.g., medical record number) allows for linkage with other data sources
Clinical signs and symptoms		
Substances for which mother/infant tested positive		
Maternal use of medication-assisted treatment		
Maternal history of substance misuse		
**Reporting system**		
State had an existing notifiable disease surveillance system	AZ, GA, VA	+ Existing in-house system allows for more rapid changes to reporting system to be implemented
		+ More timely reporting
		– Obstetric and neonatal providers might not be familiar with case reporting because many notifiable conditions are for infectious diseases
State has hospital discharge data linked to vital records	FL	+ Ability to link to other vital records and public health surveillance systems
		+ Feasible in the absence of funding resources
		– Coding errors
		– Might not capture infants delivered or treated outside of a hospital setting
		– Does not consistently capture specific substance exposures
		– Duplications in reported cases if infant is transferred
		– Deidentified data does not allow for referrals to services
State has NAS-specific reporting system	KY, TN, VA	+ Might allow for online case reporting
		+ Case report form can be easily modified
		+ Reduces need for additional resources required by paper-based system (e.g., data entry)
		– Online reporting system might require system maintenance
**Data quality**		
Data completeness	FL, GA, KY	+ Required reporting elements can reduce number of missing values
		– Delays in laboratory reports can lead to missing toxicology data
		– Lack of clinical case definition can lead to differences in variables reported by provider
**Required resources**		
Educating providers/hospitals about reporting requirements	GA, KY, TN, VA	– Added responsibility for medical provider and hospital staff members
Collecting missing data	AZ, GA	– Requires fiscal and human resources to collect missing data and to train staff members to input data and review records
Other	FL, KY	– Requires fiscal and human resources
Data cleaning		
Data reporting		
**Data utilization**		
Identification of women with substance use disorder	AZ	+ Opportunity to link women to treatment
Identification of mothers with multiple pregnancies affected by opioid exposure	FL	+ Opportunity for prevention of future NAS cases
Shared with other state and local agencies	GA, FL, KY, TN	+ Informs community assessments, planning, and program development
		+ Opportunity to evaluate the incidence of NAS within the state
		+ Informs interventions
Public reporting (as of March 2018)	AZ, GA, KY, TN	+ Opportunity to inform partners
**Barriers to case reporting**		
Limited awareness of mandate	GA	– Underreporting from providers might underestimate incidence of NAS
Limitations at the hospital/provider level	AZ, GA, KY, TN, VA	– Hospital staff member turnover can create reporting gaps/underreporting
		– Training new staff members in reporting process
		– Providers might have limited knowledge of reporting criteria
		– Complexity of reporting form

**Abbreviations**: AZ = Arizona; FL = Florida; GA = Georgia; KY = Kentucky; TN = Tennessee; VA = Virginia.

* During interviews the benefits of having a clinical diagnosis by a medical provider as part of the case definition were not specifically discussed.

^†^ In Georgia, infants with a clinical diagnosis of NAS or a positive toxicology result should be reported to the state health department.

^§^ Toxicology results do not provide information on whether a prescribed substance was used as prescribed or diverted.

Although specific approaches varied, most of the surveyed states implemented electronic reporting of NAS, which was reported as an advantage by state officials. Another resource advantage noted by state officials in Arizona and Georgia was adding NAS case reporting to existing electronic disease surveillance systems. The Tennessee and Virginia SHDs established new electronic NAS case reporting systems, and the Kentucky SHD used paper-based case report forms with plans to transition to an electronic reporting system. Florida’s passive electronic case reporting via administrative data sets did not require any changes.

Georgia, Kentucky, Tennessee, and Virginia reported that education of providers and hospital staff members on NAS case reporting requirements is one of the more resource-intensive activities related to NAS case reporting (Table 2). Arizona reported collecting missing data and training staff members on data entry and record review as challenges that require additional staffing resources. Other challenges reported by state officials include staff member turnover at hospitals and birthing centers, which could result in gaps in reporting, and the requirement that all facilities that provide care to an infant with NAS have to report the case, which poses the potential for duplicate reporting if an infant is transferred to another facility.

The numbers and types of data elements required for case reporting differed by state (Table 2). All six states collect infant demographics; Florida, Georgia, Kentucky, and Tennessee also collect maternal demographics. In addition, surveillance data were used differently by the states. Arizona, Georgia, Kentucky, and Tennessee publicly report deidentified data to inform partners and stakeholders of NAS incidence. These four states also share data with other state and local agencies to inform community assessments, planning, program development, and to provide opportunities for intervention. Arizona reported that NAS surveillance improves the state’s understanding of the proportion of NAS cases attributable to medically supervised opioid treatment during pregnancy, including pain management and medication-assisted treatment for opioid use disorder, and provides an opportunity to improve treatment strategies for pregnant women with opioid use disorder. Florida links infant and maternal hospital discharge data to connect women who have had two or more opioid-exposed pregnancies to treatment services; other states use data to promote and develop supportive care and integrated services for families.

## Discussion

This review of the six identified states’ NAS reporting laws, data collection, state officials’ reports of data quality, and data utilization identified important considerations for implementing state-based NAS surveillance. Among the six identified states that legislatively mandated reporting of NAS to SHD for public health surveillance during 2013–2017, differences in case definition and specificity of required data elements might affect the data available for monitoring and public health response. 

Since this analysis, the Council of State and Territorial Epidemiologists has convened a workgroup to develop a position statement on a standardized surveillance case definition for NAS surveillance that will be presented to the council in the summer of 2019. This will be helpful because surveyed state officials noted that the absence of a standardized NAS case definition introduces substantial variability in the type and number of cases reported to SHDs. For example, only Georgia’s NAS case definition includes asymptomatic infants with positive toxicology tests to be reported to the SHD. All surveyed states favored an electronic system for case reporting. Both benefits and limitations were noted when adapting existing electronic reporting systems or when a NAS-specific system was created de novo.

The findings in this report are subject to at least four limitations. First, narrow search terms were applied to identify laws (codified statutes and regulations) mandating NAS case reporting, which might have failed to identify states that used different terminology, mechanisms, or laws enacted since January 3, 2018. Second, four of the six laws reviewed were enacted in 2017, limiting states’ abilities to report on advantages and challenges and limiting opportunities to evaluate changes in NAS case reporting before and after laws were implemented. Third, the semistructured interview asked state informants to share areas for improvement in their case reporting systems but did not ask states to discuss perceived benefits of using a clinical diagnosis of NAS as a surveillance case definition. Finally, this report relied on qualitative data and cannot quantify the impact of these laws in states’ responses to increasing rates of NAS.

Mandated NAS case reporting might improve states’ ability to calculate more timely estimates of the incidence of NAS in their jurisdictions, identify opportunities for prevention, and facilitate linkages to care for infants and mothers. With more accurate and timely estimates of disease incidence, health systems and health care providers might be better prepared to ensure that adequate resources exist to address the immediate and potential long-term needs of children born with NAS and mothers. A standardized case definition for NAS and consistent reporting approaches will improve the ability to make meaningful comparisons between states and target prevention efforts to areas of greatest need.

**Summary**

**What is already known about this topic?**

In 2014, in the United States, an infant with neonatal abstinence syndrome (NAS) was born every 15 minutes. Historically, NAS surveillance has depended on hospital discharge data, frequently with a time lag, limiting ability to rapidly direct public health resources.

**What is added by this report?**

Among six identified states with mandated NAS reporting laws during 2013–2017, NAS incidence could be quantified to inform programs and services. However, differences in reporting methods and case definitions might influence states’ abilities to monitor NAS incidence.

**What are the implications for public health practice?**

States considering requiring NAS case reporting for public health surveillance can benefit from understanding advantages and challenges of approaches used by states with mandated NAS reporting.

## Footnotes

* https://next.westlaw.com.
